# High-Throughput Synthesis of Liposome Using an Injection-Molded Plastic Micro-Fluidic Device

**DOI:** 10.3390/mi12020170

**Published:** 2021-02-09

**Authors:** Sang-Won Woo, Yun Kyong Jo, Yeong-Eun Yoo, Sun Kyoung Kim

**Affiliations:** 1 Korea Institute of Machinery and Materials, 156 Gajeongbuk-ro, Yusung-Gu, Daejeon 34103, Korea; idolwon@gmail.com; 2 Department of Mechanical System Design Engineering, Seoul National University of Science and Technology, 232 Gongreung-Ro, Nowon-Gu, Seoul 01811, Korea; 3 Neo Nanotech Co., Ltd., Suite 304, 8-dong, 156 Gajeongbuk-ro, Yusung-Gu, Daejeon 34103, Korea; bluekyon20@naver.com

**Keywords:** liposome, micro-nozzle array, micro-channel, injection molding, plastic

## Abstract

For mass production of liposomes, we designed a plastic micro-channel device on the basis of 5 μm of micro-nozzle array forming T-junction with 100 μm depth of micro-channel. A micro-channel unit for synthesizing liposomes consisted of two micro-nozzle arrays for mixing two solutions as well as delivery and recovery channels for supplying solutions and collecting liposome suspension. The number of micro-nozzles was approximately 2400 for a micro-channel unit, and seven units were applied independently on a micro-channel plate. The plastic micro-channel plate was injection-molded for mass production using a micro-channel stamper previously fabricated by UV lithography and nickel electroforming process. A plastic cover plate with seven pairs of inlet and outlet ports was machined by mechanical milling and drilling and was assembled with a micro-channel plate using a holder to form a liposome synthesizing device. Flow and mixing of solutions in the micro-channels were tested using colored water to check the micro-fluidic characteristics of the device. Finally, a L-α-phosphatidylcholine (SOY PC) liposome was synthesized using EtOH solution of SOY PC (95%) and saline (0.85% NaOH solution) to find that the liposomes were around 230 and 260 nm in diameter, depending on the flow rate of the lipid solution.

## 1. Introduction

**Figure 1:**
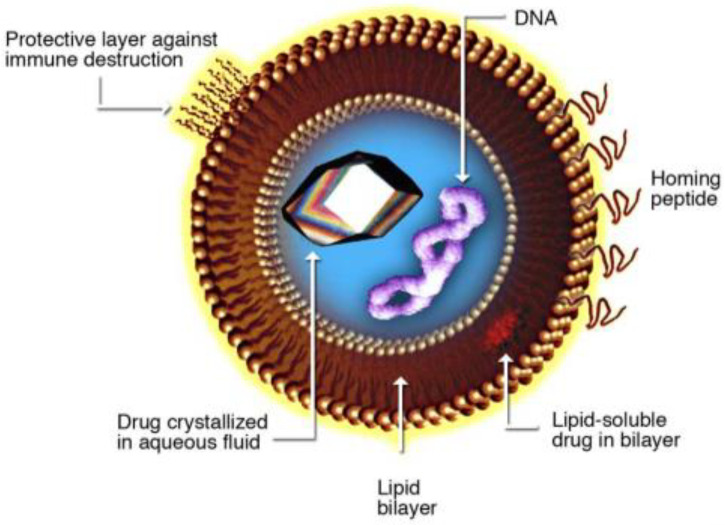
Schematic of liposome.

Liposome is a spherical lipid bilayer (Figure 1) that can encapsulate liquid containing various functional substances such as drugs, proteins, and nutrients [1]. Size of the liposome ranges from several tens of nanometers to several micrometers depending on the lipid, buffer solution, and process conditions such as flow rate of solution. Due to the good protective and controlling ability of the functional substance contained within, liposome is more widely used for applications in drug delivery [2,3,4,5,6,7], health supplements [8,9], cosmetics [10,11], oral vaccine [12,13], and biosensors [14].

To synthesize liposome, several bulk synthesis methods are used, such as ultrasonic emulsification [15], extrusion [16,17,18], and microfluidizer [19]. However, liposome synthesized by these bulk processes is generally larger than several hundreds of nanometers and shows a relatively broad size distribution. Smaller and more uniformly sized liposome has been synthesized successfully using various types of micro-fluidic channels such as T-junction channels, mostly in small amounts [20,21]. Although liposome synthesis using such channels shows outstanding performance regarding uniformity and control of size, the low throughput makes it difficult for industrial use. To solve this issue, researchers conducted studies on liposome synthesis using micro-pore membrane as a micro-fluidic platform, showing good productivity due to the large number of micro-pores on the membrane and parallel synthesizing through each pore [22,23].

In this study, a micro-channel device was designed on the basis of T-junction type of micro-channel array for scalable production of liposome. The micro-channel plate was injection-molded using thermoplastic for mass production, mechanical strength, and dimensional stability. The injection-molded micro-channel plate was sealed through mechanical assembly using a plastic cover plate with inlet and outlet ports. Using the micro-channel device, we synthesized L-α-phosphatidylcholine (SOY PC) liposomes; afterwards, their size and distribution were measured.

## 2. Design and Fabrication of a Micro-Channel Device for Synthesizing Liposome

### 2.1. Design for Micro-Channel Device

**Figure 2:**
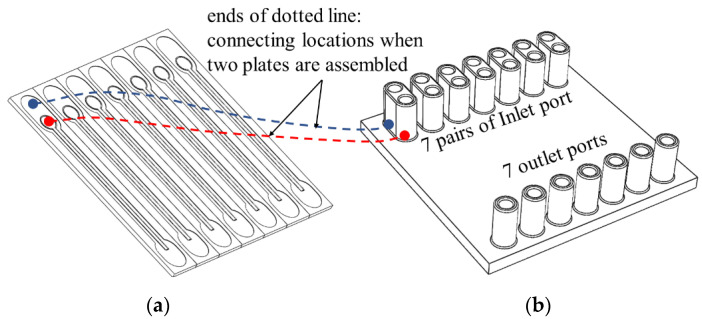
Parts for liposome synthesizing micro-channel device: (**a**) micro-channel plate and (**b**) cover plate.

**Figure 3:**
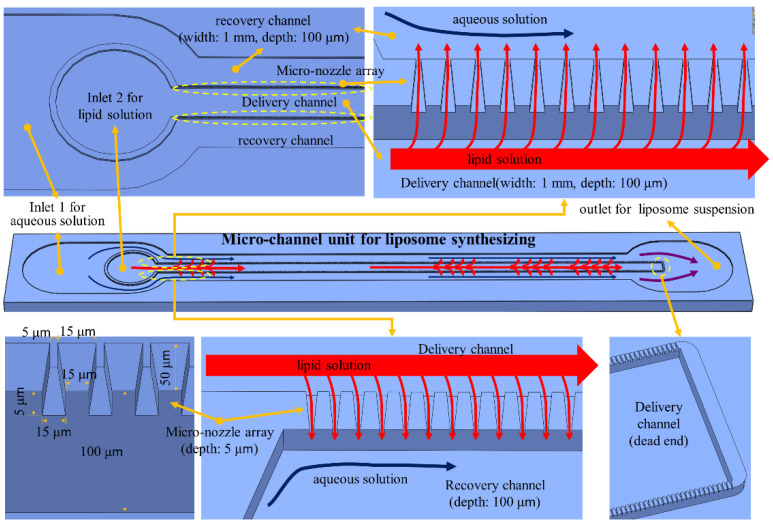
Schematics and design for a unit liposome-synthesizing micro-channel.

The micro-channel device for synthesizing liposome consists of a micro-channel plate and a cover plate with inlet and outlet ports (Figure 2). A micro-channel unit for liposome synthesis was designed to include a micro-nozzle array, a delivery channel, and two recovery channels (Figure 3). The delivery channel to deliver lipid solution is placed at the center and connected to two micro-nozzle arrays on both sides. To form multiple micro-T junctions, each micro-nozzle array connects outside with a recovery channel to supply aqueous solution for mixing with lipid solution passing through micro-nozzles from the delivery channel and to collect liposome suspension of the mixture at the same time. Two recovery channels merge into a single port to collect liposome suspension into a bottle through a tube. In this design, the lipid solution can only be mixed with the aqueous solution by passing through micro-nozzles after the delivery channel delivers it. A 37 mm long linear micro-nozzle array contains approximately 1220 micro-nozzles that gradually taper towards the exit. The dimension of a micro-nozzle is 15 μm and 5 μm in width at entrance and exit, respectively, and 50 μm in length and 5 μm in height. The delivery channel and recovery channel were designed to be 1 mm in width and 100 μm in depth to reduce pressure loss along the channel.

As shown in Figure 2, seven liposome synthesizing micro-channel units were applied to a micro-channel plate, which is 50 mm × 50 mm in size and 2 mm in thickness. The cover plate has seven pairs of ports connecting to delivery channel and recovery channel. Each pair of ports consists of two inlet ports for supplying lipid solution and aqueous solution each, and one outlet port for collecting liposome suspension.

### 2.2. Fabrication of the Plastic Device

**Figure 4:**
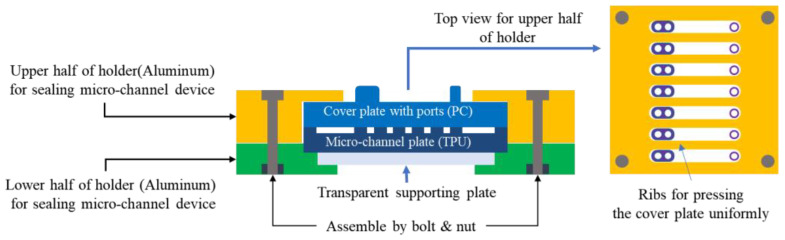
Schematic of a holder for assembling a micro-channel plate and a cover plate.

First, two parts of the liposome-synthesizing device, a micro-channel plate, and a cover plate, as shown in Figure 2, were injection-molded using thermoplastic resin or machined mechanically on plastic plate; details on the stamper fabrication process, injection molding conditions, mechanical machining by milling and drilling, and plastic materials used for the parts are explained in Section 2.3 and Section 2.4. Two plates were assembled tightly using a holder to seal the micro-channels on the micro-channel plate with the cover plate (Figure 4). The micro-channel plate was injection-molded using a nickel stamper, fabricated as explained in the following section because the micro-nozzles cannot be machined mechanically due to their size. Moreover, injection molding is suitable for mass production of micro-channel plate.

### 2.3. Fabrication of Micro-Channel Stamper

**Figure 5:**
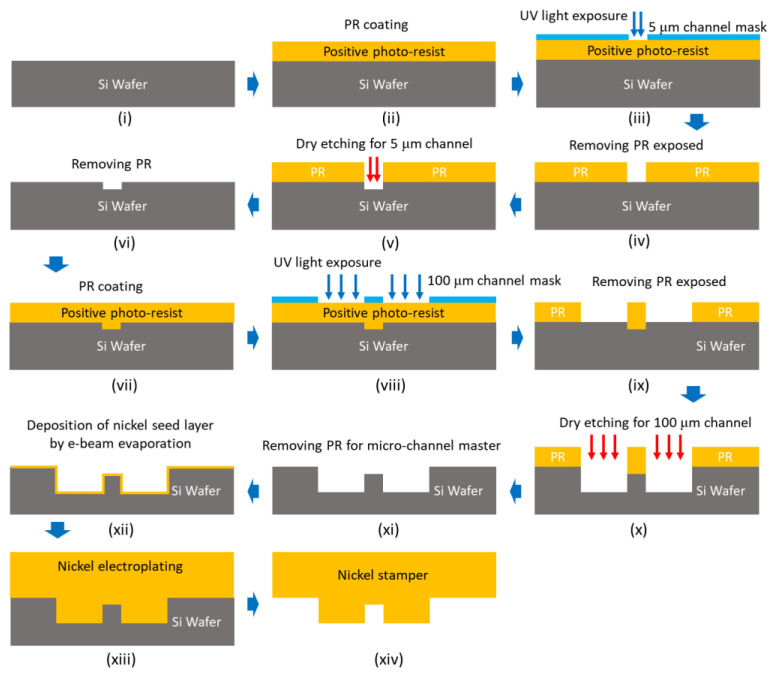
Fabrication procedure for nickel stamper including photo lithography and electroforming.

**Figure 6:**
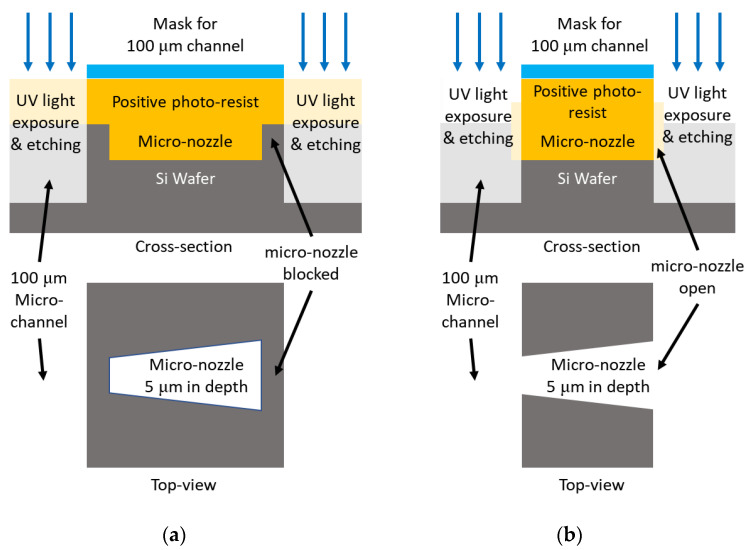
Schematic for (**a**) micro-nozzle blocked due to misalignment of mask and (**b**) design for second mask.

For injection molding of micro-channel plate, we fabricated a durable nickel stamper to be 0.5 mm in thickness and to have negative structure for micro-channel based on UV lithography process on silicon wafer and nickel electroforming process. The overall fabrication process for nickel stamper is shown in Figure 5. To fabricate stamper with channels of different depths, 5 μm-deep micro-nozzles, and 100 μm-deep delivery and recovery channel, we first fabricated silicon pattern master by repeating photo-resisting patterning and etching process twice, each using a different pattern mask, shown in steps (iii) and (viii) in Figure 5. Misalignment of these two masks can cause disconnection between the micro-nozzles and delivery or recovery channel, as shown in Figure 6a. To prevent this, we designed the masking region of the second mask aligned to the micro-nozzle array that was previously machined during steps (iii)–(vi) to be shorter by 5 μm than the length of the micro-nozzle to ensure the ends of the micro-nozzle were open, as shown in Figure 6b.

**Figure 7:**
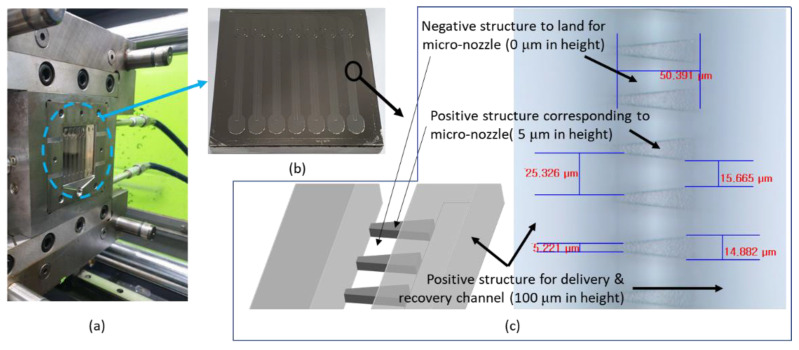
Images for (**a**) half of mold with an insert for injection molding, (**b**) micro-channel stamper insert, and (**c**) micro-channels and micro-nozzles on the stamper.

As shown in Figure 7, a nickel stamper was welded on a metal block, one of the parts for injection-molding mold. The microscopic image shows relief structures on the nickel stamper, which correspond to micro-nozzles on injection-molded micro-channel plate, and the regions out of focus correspond to delivery and recovery channel or lands in micro-nozzle array.

### 2.4. Injection Molding of Plastic Micro-Channel Plate and Mechanical Machining of Cover Plate

After mounting the nickel stamper block on the wall of the mold cavity, as shown in Figure 7, we repeatedly injection-molded the micro-channel plate using thermoplastic urethane (TPU, Lubrizol TPU-195A, Wickliffe, OH, USA), one of the thermoplastic elastomers. For injection-molding of TPU micro-channel plate, plasticizing temperature and mold temperature were 210 °C and 30 °C, respectively. The injection rate was 60 mm/s, and packing pressure of 900 kgf/cm^2^ was applied first for 1 s and then 800 kgf/cm^2^ for 2.5 s.

**Figure 8:**
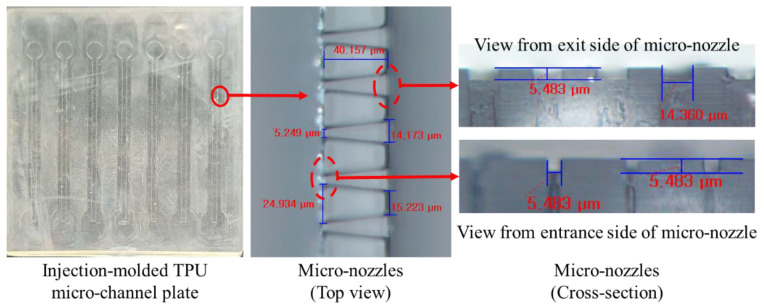
Photo for an injection-molded thermoplastic urethane (TPU) micro-channel plate and microscopic images for micro-nozzles.

Figure 8 shows the top and cross-sections of injection-molded micro-channel plate and microscopic image micro-nozzles. From the microscopic image, we can see that the micro-nozzle was injection-molded well to the corner of the land of micro-nozzle. The cycle time for micro-channel plate was less than 30 s, which is fast enough for mass production.

**Figure 9:**
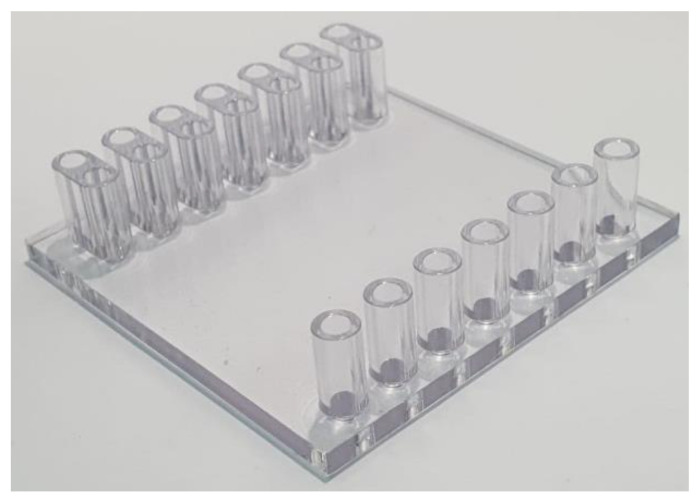
A cover plate with inlet and outlet ports machined by mechanical milling and drilling using polycarbonate block.

As shown in Figure 9, the cover plate with inlet and outlet ports was machined by mechanical milling and drilling using polycarbonate workpiece, since the structures for cover plate are good enough to be machined directly. The cover plate can also be injection-molded easily for mass production.

### 2.5. Sealing of Micro-Channel Device

**Figure 10:**
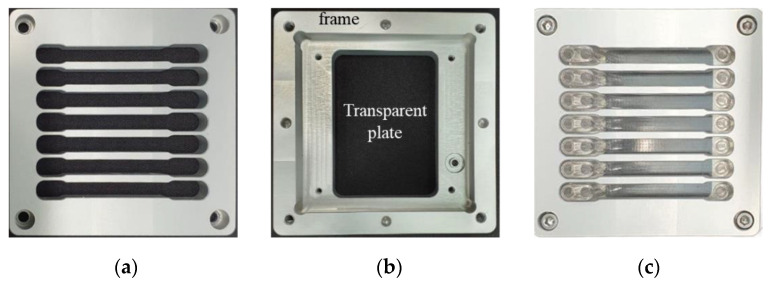
Aluminum holder machined mechanically for micro-channel device: (**a**) upper half of holder, (**b**) lower half of holder, and (**c**) assembly of holder and micro-channel device.

For sealing micro-channels and making the micro-channel device work, we stacked the micro-channel plate and cover plate and inserted them between two halves of an aluminum holder assembled by bolting to press the plates tightly (Figure 4). As seen in Figure 10a, the upper half of the holder has ribs to press the cover plate with ports more evenly. The lower half of the holder presses the micro-channel plate injection molded with TPU, which is deformed easily and therefore is good for sealing of micro-channels when covered with a rigid plate. The lower half of the holder consists of a rigid outer frame and a transparent plate for monitoring the flow in the channel (Figure 10b). The transparent plate also supports the channel plate to prevent deformation or bending when the channel plate and cover plate are assembled tightly, which may result in leakage from the channels.

## 3. Mixing Fluids and Synthesizing Liposome Using Micro-Channel Device

### 3.1. Test for Mixing of Two Fluids in Micro-Channel Device

**Figure 11:**
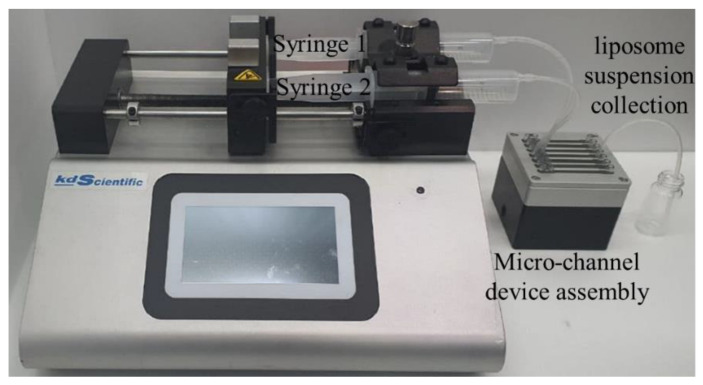
Experiment set-up for flow and mixing test or synthesizing liposome using micro-channel device.

For testing flow and mixing of fluids through micro-nozzle and channels, we used two types of water, one yellow and the other blue, for visualization of flow and mixing. The yellow water was supplied to the delivery channel through the inlet port for lipid solution (inlet port 2 in Figure 3) and flowed into recovery channel through the micro-nozzle to mix with the blue water supplied to the recovery channel through inlet port for buffer solution (inlet port 1 in Figure 3). The flow rate for each fluid was controlled independently using a syringe pump, as shown in Figure 11.

**Figure 12:**
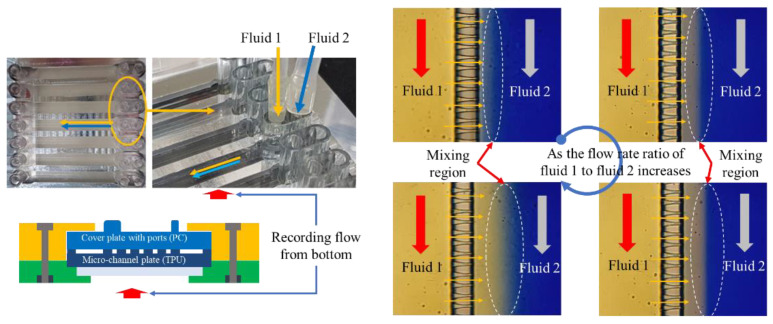
Flow and mixing visualization in micro-channel device.

When we supplied yellow and blue water to the inlet ports, some microscopic images were taken on interfacial regions of yellow and blue water along the recovery channel where two fluids mixed, with the neighboring region including micro-nozzle array and delivery channel (Figure 12). The interface between two fluids was formed parallel to the recovery channel due to the flow injected orthogonally from micro-nozzles arrayed closely in a row. Depending on the flow rate of fluid through inlet 1 for lipid solution, the location of the interface moved close to the micro-nozzle array or away from the micro-nozzle array.

### 3.2. Synthesizing Liposome Using Micro-Channel Device

SOY PC (95%) (L-α-phosphatidylcholine, Avanti Polar Lipids, Inc., alabaster, AL, USA) and saline (0.85% NaOH solution) were used to synthesize liposome using micro-channel device. A total of 0.775 g of granular SOY PC was mixed with 10 mL of EtOH for 3 h using a magnetic stirrer to produce 10 mM of SOY PC EtOH solution. This SOY PC solution was supplied to the micro-channel device through inlet port 1 at two different flow rates, 3 mL/h and 4.5 mL/h. Saline, 0.85% of NaOH solution, was supplied through inlet port 2 at a 30 mL/h flow rate (Figure 3). Liposome synthesis experiments were repeated three times, each for a set of flow rate conditions using the same SOY PC EtOH solution and saline.

The liposome suspension was heated up to 80 °C for 1 h so that it could be volatilized after being collected through the outlet port from recovery channel where the two solutions were mixed. The boiled liposome suspension was cooled down to room temperature and stored in a chamber at 5 °C for 20 h. NANOPHOX (Sympatec GmbH, Clausthal-Zellerfeld, Germany), a photon cross-correlation spectroscopy (PCCS), was used to analyze the size of the liposome in the suspension.

**Figure 13:**
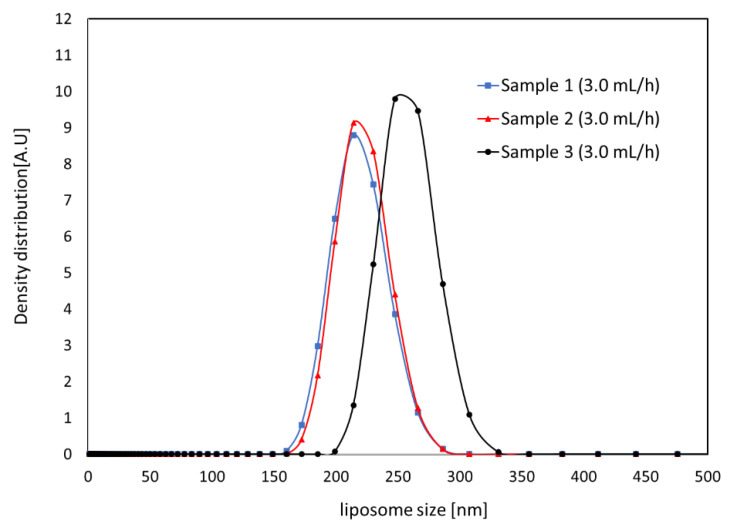
Liposome size distribution measured for three samples synthesized at 3.0 mL/h of lipid solution and 30 mL/h of saline.

**Figure 14:**
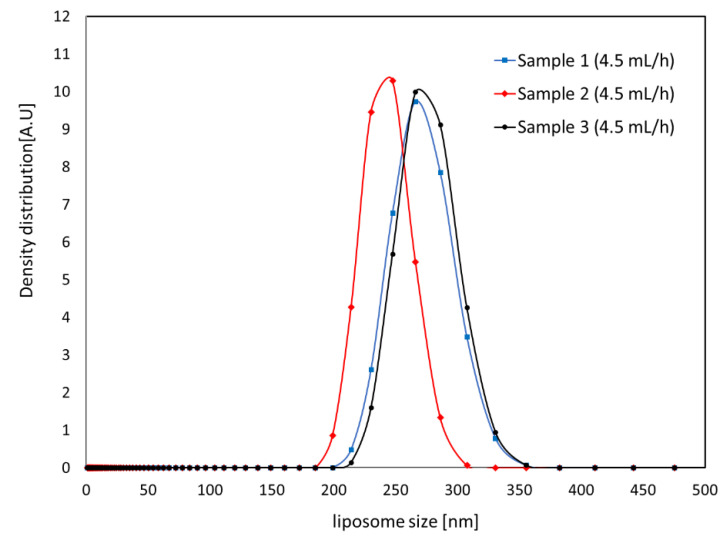
Liposome size distribution measured for three samples synthesized at 4.5 mL/h of lipid solution and 30 mL/h of saline.

The results for the measured size of the SOY PC liposomes synthesized by micro-channel device are shown in Figure 13 and Figure 14. The graph shows the intensity of the signal depending on the size of the liposome in the solution. The average diameter, $\bar{d}$, and standard deviation, σ, of the liposomes in a sample were estimated by Equation (1) and Equation (2), respectively, to observe size variation in the sample, and the results are shown in Table 1. For both flow rate conditions faster than 30 mL/h of total flow rate, liposome was smaller than 300 nm with approximately 10% of standard deviation of average size.

(1) $$\bar{d}=\;\frac{\sum \left(I_{i}d_{i}\right)}{\sum I_{i}}$$

(2) $$\sigma =\;\sqrt{\frac{\sum {\left(\bar{d}-d_{i}\right)}^{2}\;\times \;I_{i}}{\sum I_{i}}}$$

where $d_{i}$ and $I_{i}$ are diameter of liposome and signal density from spectroscopy for liposome of diameter $d_{i}$, respectively.

**Figure 15:**
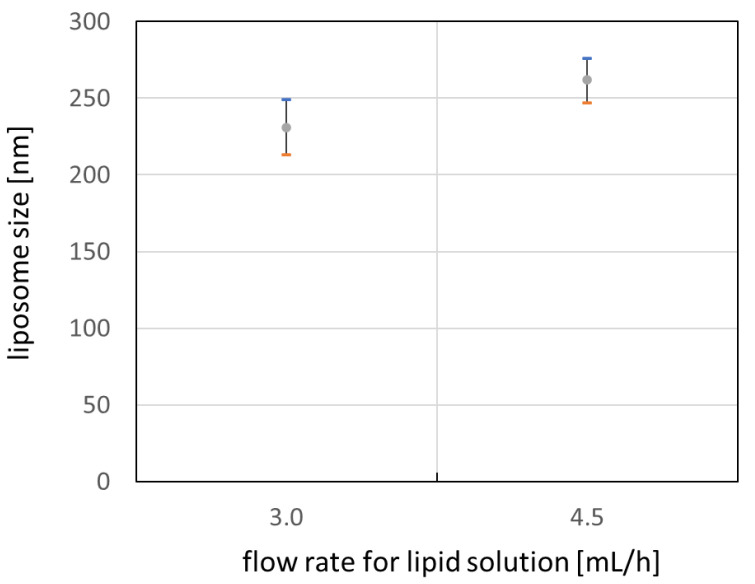
Liposome size depending on flow rate for lipid solution.

Figure 15 shows average size of liposomes for each flow rate conditions with error bar of one standard deviation. It is noticed that the size of the liposome can be controlled by flow rate of the lipid solution or buffer. As the ratio of lipid solution flow rate to the buffer flow rate increases, the characteristic time for mixing two solution increases also. This increase in mixing time may enhance the growth of the lipid bilayer and result in larger liposomes when the lipid bilayer forms a vesicle after linear growth [24,25,26]. However, extended experimental investigation may be required to figure out the effect on the size of the liposome for a wider range of flow rate conditions.

From the viewpoint of throughput, 30 mL/h of flow rate in this work is much higher than a flow rate less than 1 mL/h for typical single T-junction micro-channel [21]. This indicates that the throughput for synthesizing liposome can be enhanced greatly by T-junction array formed by micro-nozzles and recovery channel. In addition, synthesizing liposome is scalable since the throughput can be enhanced by magnitude of the number of T-junction arrays in a micro-channel device when the solutions are supplied simultaneously to all arrays at the same time.

## 4. Conclusions

A high-throughput plastic micro-channel device for liposome synthesis was designed and fabricated by injection molding or mechanical machining depending on part and assembling the parts. The micro-channel plate, a core part of the liposome synthesizing device, was injection-molded with a cycle time of less than 30 s, which is fast enough for mass production. The micro-channel device showed that fluids flowed and mixed well through micro-nozzles and micro-channels. Using the micro-channel device, we synthesized SOY PC liposome by mixing EtOH solution of SOY PC and saline (0.85% NaOH solution). The size of the liposome varied depending on the flow rate conditions of solutions. In the case of 30 mL/h of flow rate for saline, the liposome diameter was 230 nm or 260 nm for 3.0 mL/h or 4.5 mL/h SOY PC solution, respectively. The size of each liposome sample had a standard deviation of less than 20 nm, which indicates good uniformity in size.

Overall, this study implies that the throughput for synthesizing liposome can be enhanced linearly by using multiple micro-channel units. Additional extensive studies are required to figure out the precise correlation between size of the liposome and flow rate.

## Figures and Tables

**Table 1:** Average and standard deviation for liposomes.

Sample	Lipid Solution 3 mL/h, Saline 30 mL/h		Lipid Solution 4.5 mL/h, Saline 30 mL/h	
	Average Size (nm)	Standard Deviation	Average Size (nm)	Standard Deviation
I	217	22	270	25
II	220	21	242	20
III	257	22	274	24
